# Distinct effects of cholesterol profile components on amyloid and vascular burdens

**DOI:** 10.1186/s13195-023-01342-2

**Published:** 2023-11-10

**Authors:** Sung Hoon Kang, Heejin Yoo, Bo Kyoung Cheon, Yu Hyun Park, Soo-Jong Kim, Hongki Ham, Hyemin Jang, Hee Jin Kim, Kyungmi Oh, Seong-Beom Koh, Duk L. Na, Jun Pyo Kim, Sang Won Seo

**Affiliations:** 1 Department of Neurology, Sungkyunkwan University School of Medicine, Samsung Medical Center, 81 Irwon-ro, Gangnam-gu, Seoul, 06351 Korea; 2 Department of Neurology, Korea University Guro Hospital, Korea University College of Medicine, Seoul, Korea; 3 Alzheimer’s Disease Convergence Research Center, Samsung Medical Center, Seoul, Korea; 4 Department of Digital Health, SAIHST, Sungkyunkwan University, Seoul, Korea; 5 Department of Intelligent Precision Healthcare Convergence, Sungkyunkwan University, Suwon, Korea; 6 Department of Health Sciences and Technology, SAIHST, Sungkyunkwan University, Seoul, Korea

**Keywords:** LDL-c, HDL-c, β-Amyloid-β (Aβ), White matter hyperintensity (WMH), Hippocampal volume

## Abstract

**Background:**

Cholesterol plays important roles in β-amyloid (Aβ) metabolism and atherosclerosis. However, the relationships of plasma cholesterol levels with Aβ and cerebral small vessel disease (CSVD) burdens are not fully understood in Asians. Herein, we investigated the relationships between plasma cholesterol profile components and Aβ and CSVD burdens in a large, non-demented Korean cohort.

**Methods:**

We enrolled 1,175 non-demented participants (456 with unimpaired cognition [CU] and 719 with mild cognitive impairment [MCI]) aged ≥ 45 years who underwent Aβ PET at the Samsung Medical Center in Korea. We performed linear regression analyses with each cholesterol (low-density lipoprotein cholesterol [LDL-c], high-density lipoprotein cholesterol [HDL-c], and triglyceride) level as a predictor and each image marker (Aβ uptake on PET, white matter hyperintensity [WMH] volume, and hippocampal volume) as an outcome after controlling for potential confounders.

**Results:**

Increased LDL-c levels (β = 0.014 to 0.115, *p* = 0.013) were associated with greater Aβ uptake, independent of the *APOE* e4 allele genotype and lipid-lowering medication. Decreased HDL-c levels (β =  − 0.133 to − 0.006, *p* = 0.032) were predictive of higher WMH volumes. Increased LDL-c levels were also associated with decreased hippocampal volume (direct effect β =  − 0.053, *p* = 0.040), which was partially mediated by Aβ uptake (indirect effect β =  − 0.018, *p* = 0.006).

**Conclusions:**

Our findings highlight that increased LDL-c and decreased HDL-c levels are important risk factors for Aβ and CSVD burdens, respectively. Furthermore, considering that plasma cholesterol profile components are potentially modified by diet, exercise, and pharmacological agents, our results provide evidence that regulating LDL-c and HDL-c levels is a potential strategy to prevent dementia.

## Background

Alzheimer’s disease (AD) and cerebral small vessel disease (CSVD) are common causes of dementia in the elderly. AD is characterized by the deposition of β-amyloid (Aβ), which starts to accumulate 10–20 years before the onset of clinical symptoms. CSVD burdens, such as severe white matter hyperintensities (WMH), are also gradually deposited in subcortical regions over several decades, eventually resulting in subcortical vascular dementia. Recent advances in neuroimaging have enabled the detection of these pathological burdens in living individuals without dementia.

Plasma cholesterol levels are major risk factors for coronary artery disease and stroke. Plasma cholesterol levels are also closely associated with other components of the cardiometabolic syndrome, which may affect the development of dementia [1, 2]. However, epidemiological evidence of the association between plasma cholesterol levels and dementia is complex [3]. Some studies have shown that increased total cholesterol levels are predictive of an increased risk of dementia. In contrast, other studies have not shown a relationship between total cholesterol levels and the risk of dementia. In fact, other studies have reported that increased total cholesterol levels are associated with a reduced risk of dementia. These inconsistencies in the findings may be explained by several factors. First, most previous studies have not investigated the relationship between plasma cholesterol levels and biomarkers of AD and CSVD. The clinical diagnostic criteria for AD and CSVD do not necessarily represent the presence of AD and CSVD imaging markers. Moreover, most studies were limited to total cholesterol rather than dysregulation of cholesterol homeostasis, such as increased low-density lipoprotein cholesterol (LDL-c) or decreased high-density lipoprotein cholesterol (HDL-c). Dysregulation of cholesterol homeostasis is a more important risk factor for cardiovascular disease than total cholesterol levels are.

Cholesterol in the brain plays fundamental roles in the synthesis and maintenance of neuronal cells and is linked to brain health [4]. Cholesterol has also important roles in the synthesis, deposition, and clearance of Aβ [5, 6]. Plasma cholesterol levels are well-known atherogenic risk scores. Several studies have investigated the relationships between plasma cholesterol profile components and biomarkers [7–9]. However, the results of these studies were inconclusive because the sample sizes were small. In addition, racial diversity was lacking among the participants. In fact, differences exist in the body composition, effects of cardiometabolic syndromes on brain age, and frequencies of imaging marker abnormalities among Asians and non-Hispanic whites (NHWs). Specifically, the Asian population has a higher risk of hypercholesterolemia [10] and cardiometabolic syndromes [11] than NHWs with a similar body mass index (BMI), which is explained by unfavorable body composition and fat distribution [12, 13]. In addition, the Asian population has higher CSVD burdens and a lower Aβ frequency than NHWs [14, 15].

Therefore, in the present study, we investigated the relationships between plasma cholesterol profile components and biomarkers of AD and CSVD in a large non-demented Korean cohort. To avoid the potential reverse effects of dementia on blood cholesterol, participants were limited to non-demented participants, although participants with mild cognitive impairment (MCI) might be more likely to have poor cholesterol profile than those with unimpaired cognition (CU). We hypothesized that increased LDL-c and decreased HDL-c levels would be predictive of increased Aβ and CSVD burdens. Furthermore, these imaging markers may mediate the relationship between plasma cholesterol profile components and downstream imaging markers, including hippocampal atrophy.

## Methods

### Study participants

We enrolled 1,175 non-demented participants (456 with CU and 719 with MCI) aged ≥ 45 years who underwent Aβ PET in the memory clinic at the Samsung Medical Center (Seoul, Korea) between August 2015 and August 2020. All participants underwent a comprehensive dementia examination including a standardized neuropsychological test [16], *APOE* genotyping, and brain magnetic resonance imaging (MRI). All participants with CU met the following criteria: (1) no medical history that was likely to affect cognitive function based on Christensen’s health screening criteria [17]; (2) no objective cognitive impairment in any cognitive domain on a comprehensive neuropsychological test battery (above at least -1.0 SD of age-adjusted norms on any cognitive test); and (3) independence in activities of daily living. All participants with MCI met the criteria for MCI with the following modifications [18, 19]: (1) subjective cognitive complaints by the participants or caregivers; (2) objective memory impairment below -1·0 SD on verbal or visual memory tests; (3) no significant impairment in activities of daily living; and (4) non-demented status.

We excluded participants with severe WMH (cap or band > 10 mm and longest diameter of deep white matter lesion > 25 mm), structural lesions including cerebral infarction, intracranial hemorrhage, brain tumors, and hydrocephalus on MRI, and abnormal laboratory results on complete blood count, electrolyte, vitamin B12 and folate levels, syphilis serology, and liver/kidney/thyroid function tests.

The Institutional Review Board of the Samsung Medical Center approved this study. Written informed consent was obtained from all the participants.

### Aβ PET acquisition

All participants underwent Aβ PET (^18^F-florbetaben) and ^18^F-flutemetamol PET scans using a Discovery STe PET/CT scanner (GE Medical Systems, Milwaukee, WI, USA). For ^18^F-florbetaben PET or ^18^F-flutemetamol PET, a 20-min emission PET scan in dynamic mode (comprising 4 × 5 min frames) was performed 90 min after an injection of a mean dose of 311.5 MBq ^18^F-florbetaben or 197.7 MBq ^18^F-flutemetamol, respectively. Three-dimensional PET images were reconstructed in a 128 × 128 × 48 matrix with 2 × 2 × 3·27 mm voxel size using the ordered-subsets expectation maximization algorithm (^18^F-florbetaben, iteration = 4 and subset = 20; ^18^F-flutemetamol, iteration = 4 and subset = 20).

### Aβ PET quantification using dcCL scales

Aβ uptakes were quantified using BeauBrain Morph of BeauBrain Healthcare Co., Ltd., which performs fully-automated image analysis of Aβ uptakes on PET images. We used a direct comparison of the FBB-FMM CL (dcCL) method previously developed by our group [20] to standardize the quantification of Aβ PET images obtained using different ligands. The dcCL method for FBB and FMM PET enables the transformation of the standardized uptake value ratio (dcSUVR) of FBB and FMM PETs to dcCL scales directly, without conversion to the ^11^C-labeled Pittsburgh compound SUVR.

There are three steps to obtaining dcCL scales [20]: 1) pre-processing of PET images, 2) determination of the global cortical target volume of interest (CTX VOI), and 3) conversion of dcSUVR to dcCL scales. First, to preprocess the Aβ PET images, PET images were co-registered to each participant’s MR image and then normalized to a T1-weighted MNI-152 template using the SPM8 unified segmentation method. We used T1-weighted MRI correction with the N3 algorithm only for intensity nonuniformities without applying corrections to the PET images for brain atrophy or partial volume effects. Second, we used the FBB-FMM CTX VOI, defined as areas of AD-specific brain Aβ deposition in our previous study [20]. In our previous study [20], we developed CTX VOI using the similar methods with Klunk’s Centiloid methods [21]. In their original study [21], they included 19 patients with AD dementia and 25 older controls to determine areas of AD-specific brain Aβ deposition. Briefly, to exclude areas of age-related brain Aβ deposition, the FBB-FMM CTX VOI was generated by comparing SUVR parametric images (with the whole cerebellum as a reference area) between 20 typical patients with Alzheimer’s disease-related cognitive impairment (ADCI-CTX) and 16 healthy elderly participants (EH-CTX) who underwent both FBB and FMM PET scans. To generate the FBB-FMM CTX VOI, the average EH-CTX image is subtracted from the average ADCI-CTX image. We then defined the FBB-FMM CTX VOI as the area of ADCI-related brain Aβ accumulation common to both FBB and FMM PET. Finally, the dcSUVR values of the FBB-FMM CTX VOI were converted to dcCL scales by using the dcCL conversion equation. The dcCL equation was derived from the FBB-FMM CTX VOI separately for FBB and FMM PET, and applied to FBB and FMM dcSUVR.

To determine the participants’ dcCL cut-off-based Aβ positivity, we applied the optimal cut-off value derived using *k*-means cluster analysis in 527 independent samples of participants with normal cognition. The cut-off value was set at 27.08, representing the 95^th^ percentile of the lower cluster, and the whole cerebellum was used as a reference region.

### Cholesterol and other covariate measurements

All participants underwent blood tests. Blood samples were collected after an overnight fast. The mean ± SD of time interval between blood tests and Aβ PET was 3.9 ± 5.0 months. Cholesterol levels, including LDL-c, HDL-c, and triglycerides, were measured by an enzymatic colorimetric test using a Modular D2400 analyzer (Roche Diagnostics, Basel, Switzerland). Height and weight were measured for all participants. BMI was calculated as weight (kg) divided by the square of height (m). Cholesterol levels and BMI data were obtained by backtracking in the clinical data warehouse (CDW) of the Samsung Medical Center, which were measured within 12 months before or after Aβ PET scans. Prescriptions of lipid-lowering and dementia medications (donepezil, rivastigmine, galantamine, or memantine) were extracted from the CDW. Hypertension and diabetes were defined as a diagnostic history of hypertension and diabetes or current use of any antihypertensive medication and antidiabetic medication, respectively.

### MRI acquisition

We acquired standardized three-dimensional T1 Turbo Field Echo and three-dimensional fluid-attenuated inversion recovery (FLAIR) images using a 3.0 T MRI scanner (Philips 3.0T Achieva; Philips Healthcare, Andover, MA, USA), as previously described [22].

### Hippocampal and WMH volume

The images were processed using the CIVET anatomical pipeline (version 2.1.0). The native MRIs were registered to the MNI-152 template by linear transformation and corrected for intensity nonuniformities using the N3 algorithm. The registered and corrected images were divided into white matter, grey matter, cerebrospinal fluid, and background. In addition, the inner and outer surfaces of the cortex were automatically extracted using the marching-cubes algorithm to obtain the cortical thickness, which was defined as the Euclidean distance between the linked vertices of the inner and outer surfaces.

As we extracted cortical surface models from MRI volumes transformed into stereotaxic space, the cortical thickness was measured in the native space by applying an inverse transformation matrix to the cortical surface and reconstructing them in the native space.

To measure hippocampal volume, we used an automated hippocampus segmentation method using a graph cut algorithm combined with atlas-based segmentation and morphological opening, as described in a previous study [23]. WMH segmentation was replicated using the Lesion Segmentation Tool (LST) in Statistical Parametric Mapping 12 [24]. LST is an automated segmentation approach for quantifying whole-brain WMH volume and shows high agreement with manual tracing of WMH in FLAIR.

### Statistical analyses

To investigate the association between cholesterol levels and Aβ uptake, we performed linear regression analyses with each cholesterol type (LDL-c, HDL-c, and triglyceride) as a predictor and quantified dcCL scales as an outcome after controlling for age, sex, BMI, APOE e4 allele (*APOE4*) genotype, hypertension, diabetes, lipid-lowering medication, and dementia medication.

To investigate the association between cholesterol levels and neurodegeneration, we performed linear regression analyses with each cholesterol type (LDL-c, HDL-c, and triglyceride) as a predictor and hippocampal volume as an outcome after controlling for age, sex, BMI, *APOE4* genotype, hypertension, diabetes, lipid-lowering medication, dementia medication, and intracranial volume.

To investigate the association between cholesterol levels and WMH volume, we performed linear regression analyses with each cholesterol type (LDL-c, HDL-c, and triglyceride) as a predictor and hippocampal volume as an outcome after controlling for age, sex, BMI, *APOE4* genotype, hypertension, diabetes, lipid-lowering medication, dementia medication, and intracranial volume.

To determine whether Aβ uptake mediates the effect of cholesterol levels on neurodegeneration, we used path analyses with each cholesterol type as a predictor, Aβ uptake as a mediator, and hippocampal volume as an outcome after controlling for age, sex, BMI, *APOE4* genotype, hypertension, diabetes, lipid-lowering medication, dementia medication, and intracranial volume.

The standardized β was also calculated to compare the strength of the effect of each independent variable on each dependent variable in all analyses.

Sensitivity analyses using cut-off-based categorization rather than quantified dcCL values were performed to further validate the relationship between cholesterol levels and Aβ uptake. We used logistic regression analysis with each cholesterol type (LDL-c, HDL-c, and triglyceride) as a predictor and Aβ positivity as an outcome after controlling for age, sex, BMI, *APOE4* genotype, hypertension, diabetes, lipid-lowering medication, and dementia medication. In addition, we performed a single model with all cholesterol type (LDL-c, HDL-c, and triglyceride) together as predictors after age, sex, BMI, APOE e4 allele (APOE4) genotype, hypertension, diabetes, lipid-lowering medication, and dementia medication.

All reported *p*-values were two-sided and the significance level was set at 0.05. All analyses were performed using SAS version 9.4 (SAS Institute, Inc., Cary, NC, USA).

## Results

### Clinical characteristics of the participants

The mean ± SD of age was 70.8 ± 8.2 years, and 641 out of 1125 (54.6%) participants were female (Table 1). Four hundred and forty-six out of 1125 (38.0%) patients had *APOE4* genotype. The mean ± SD of Aβ uptake was 39.8 ± 46.7 dcCL. The mean ± SD of WMH and hippocampal volume were 5.27 ± 9.33 and 2.78 ± 5.07 mL, respectively. The mean ± SD of LDL-c, HDL-c, and triglyceride were 109.2 ± 35.1, 58.6 ± 16.7, and 118.5 ± 55.9 mg/dL. There were no differences in LDL-c (107.6 ± 33.9 vs 110.2 ± 35.8, *p* = 0.208), HDL-c (59.1 ± 16.4 vs 58.3 ± 16.9, *p* = 0.382), and triglyceride (120.0 ± 53.6 vs 117.6 ± 57.3, *p* = 0.477) level between CU and MCI groups.

**Table 1:** Demographic and clinical characteristics of the study participants

	Total (*n* = 1125)	CU (*n* = 456)	MCI (*n* = 719)	*p*
**Demographics**				
Age, years	70.8 ± 8.2	70.6 ± 8.1	70.9 ± 8.2	0.557
Middle-aged, ≤ 65 years	272 (23.1%)	103 (22.6%)	169 (23.5%)	0.770
Gender, females	641 (54.6%)	268 (58.8%)	373 (51.9%)	0.024
Education, years	12.5 ± 4.7	12.2 ± 4.7	12.2 ± 4.7	0.909
**Clinical characteristics**				
APOE e4 carrier	446 (38.0%)	128 (28.1%)	318 (44.2%)	< 0.001
Hypertension	530 (45.1%)	213 (46.7%)	317 (44.1%)	0.412
Diabetes	233 (19.8%)	94 (20.6%)	139 (19.3%)	0.644
Lipid-lowering medication	368 (31.3%)	138 (30.3%)	230 (32.0%)	0.578
AChE inhibitor	647 (55.1%)	113 (24.8%)	534 (74.3%)	< 0.001
Memantine	70 (6.0%)	13 (2.9%)	57 (7.9%)	0.001
**Cholesterol**				
LDL-c	109.2 ± 35.1	107.6 ± 33.9	110.2 ± 35.8	0.208
HDL-c	58.6 ± 16.7	59.1 ± 16.4	58.3 ± 16.9	0.382
Triglyceride	118.5 ± 55.9	120.0 ± 53.6	117.6 ± 57.3	0.477
**Imaging markers**				
Aβ uptake, dcCL	39.8 ± 46.7	20.0 ± 34.6	52.3 ± 49.0	< 0.001
WMH volume, mL	5.27 ± 9.33	4.64 ± 10.26	5.69 ± 8.66	0.085
HV, mL	2.78 ± 0.5	2.98 ± 0.4	2.65 ± 0.5	< 0.001

Values are presented as mean ± SD or n (%)

*Abbreviations: AChE* Acetylcholinesterase, *CU* Cognitively unimpaired, *HDL-c* High-density lipoprotein cholesterol, *HV* Hippocampal volume, *LDL-c* Low-density lipoprotein, *MCI* Mild cognitive impairment, *WMH* White matter hyperintensity

*p*-values are obtained by independent t-tests and chi-square tests between CU and MCI groups

### Cholesterol levels and Aβ uptake

**Fig. 1:**
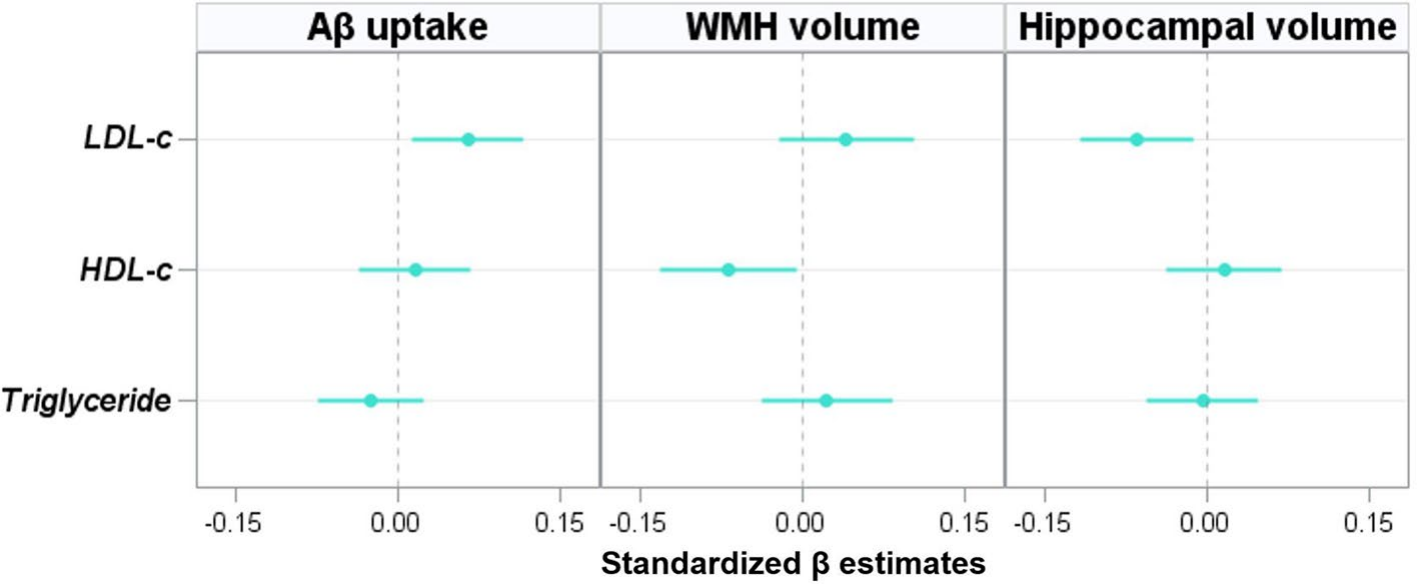
Relationship on of cholesterol levels with Aβ uptake, WMH volume and HV. General linear regression analyses were performed using each cholesterol level (LDL-c, HDL-c, and triglycerides) as predictors, and dcCL scale, WMH volume, and HV as outcomes after controlling for age, sex, BMI, *APOE4* genotype, hypertension, diabetes, lipid-lowering medication, and dementia medication. The standardized β was calculated to compare the strength of the effect of each independent variable on each dependent variable. Abbreviations: Aβ, amyloid-β; BMI, body mass index; HDL-c, high-density lipoprotein cholesterol; HV, hippocampal volume; LDL-c, low-density lipoprotein cholesterol; WMH, white matter hyperintensity

LDL-c levels (β = 0.064, *p* = 0.013) were independently associated with Aβ uptake. However, there were no relationships of HDL-c (β = 0.016, *p* = 0.552) and triglycerides levels (β =  − 0.025, *p* = 0.308) with Aβ uptake (Table 2, Fig. 1).

**Table 2:** Association of cholesterol levels with Aβ uptake, WMH volume, and HV

	Aβ uptake		WMH volume		HV	
	β (95% CI)^a^	*p*	β (95% CI)	*p*	β (95% CI)	*p*
Unstandardized						
LDL-c	0.085 (0.018, 0.153)	0.013	0.003 (-0.002, 0.009)	0.204	-0.942 (-1.693, -0.192)	0.014
HDL-c	0.043 (-0.100, 0.187)	0.552	-0.012 (-0.024, -0.001)	0.032	0.468 (-1.128, 2.064)	0.565
Triglyceride	-0.021 (-0.062, 0.020)	0.308	0.001 (-0.002, 0.004)	0.474	-0.037 (-0.501, 0.426)	0.875
Standardized^b^						
LDL-c	0.064 (0.014, 0.115)	0.013	0.040 (-0.022, 0.103)	0.204	-0.065 (-0.117, -0.013)	0.014
HDL-c	0.016 (-0.036, 0.067)	0.552	-0.069 (-0.133, -0.006)	0.032	0.015 (-0.037, 0.068)	0.565
Triglyceride	-0.025 (-0.074, 0.023)	0.308	0.022 (-0.039, 0.083)	0.474	-0.004 (-0.055, 0.047)	0.875

*Abbreviations: Aβ* Amyloid-β, *BMI* Body mass index, *CI* Confidence interval, *HDL-c* High-density lipoprotein cholesterol, *HV* Hippocampal volume, *LDL-c* Low-density lipoprotein cholesterol, *WMH* White matter hyperintensity

^a^β was obtained by general linear regression analyses with each cholesterol (LDL-c, HDL-c, and triglycerides) as a predictor and quantified dcCL scale as an outcome, after controlling for age, sex, BMI, *APOE4* genotype, hypertension, diabetes, lipid-lowering medication, and dementia medication

^b^Standardized β was calculated to compare the strength of the effect of each independent variable on each dependent variable

### Cholesterol levels and WMH volume

HDL-c levels (β =  − 0.069, *p* = 0.032) were negatively associated with WMH volume. However, LDL-c (β = 0.040, *p* = 0.204) and triglyceride (β = 0.022, *p* = 0.474) levels were not associated with WMH volume (Table 2, Fig. 1).

### Cholesterol levels and hippocampal volume

**Fig. 2:**
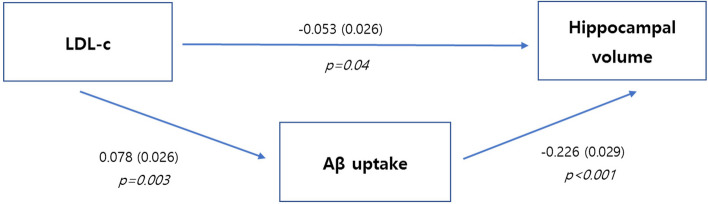
Schematic diagram of the path analyses among LDL-c, Aβ burden, and HV. Path analyses were performed using LDL-c as a predictor, Aβ uptake as a mediator, and hippocampal volume as an outcome, after controlling for age, sex, BMI, *APOE4* genotype, hypertension, diabetes, lipid-lowering medication, dementia medication, and intracranial volume. Abbreviations: HV, hippocampal volume; LDL-c, low-density lipoprotein cholesterol

LDL-c levels (β =  − 0.065, *p* = 0.008) were negatively associated with hippocampal volume, whereas HDL-c (β = 0.015, *p* = 0.565) and triglyceride (β =  − 0.004, *p* = 0.737) levels were not associated with hippocampal volume (Table 2, Fig. 1). Path analyses showed that Aβ uptake partially mediated the association between LDL-c and hippocampal volume. Specifically, increased LDL-c levels were associated with Aβ uptake (β = 0.078, *p* = 0.003), which was further associated with hippocampal volume (β =  − 0.226, *p* < 0.001; indirect pathway β =  − 0.018, *p* = 0.006). Increased LDL-c levels were also associated with hippocampal volume without mediation of Aβ uptake (direct pathway β =  − 0.053, *p* = 0.040; Table 3, Fig. 2).

**Table 3:** Effect of LDL-c levels on Aβ uptake and HV

		Unstandardized β (SE)	Standardized β (SE)	*p*
Indirect	LDL-c → Aβ → HV	-0.253 (0.092)	-0.018 (0.006)	0.006
Component	LDL-c → Aβ	0.104 (0.035)	0.078 (0.026)	0.003
	Aβ → HV	-2.436 (0.317)	-0.226 (0.029)	< 0.001
Direct	LDL-c → HV	-0.759 (0.370)	-0.053 (0.026)	0.040
Total	LDL-c → HV	-1.012 (0.379)	-0.071 (0.026)	0.007

Path analyses were performed using LDL-c as a predictor, Aβ uptake as a mediator, and hippocampal volume as an outcome, after controlling for age, sex, BMI, *APOE4* genotype, hypertension, diabetes, lipid-lowering medication, dementia medication, and intracranial volume. The standardized β was calculated to compare the strength of the effect of each independent variable on each dependent variable

### Sensitivity analysis

Regarding the categorical values of Aβ uptake, LDL-c levels (Odds ratio [OR] = 1.005, 95% confidence interval [CI] 1.001 to 1.009, *p* = 0.039) were independently associated with Aβ positivity. However, there were no relationships of HDL-c (OR = 1.002, 95% CI 0.993 to 1.012, *p* = 0.647) and triglycerides levels (OR = 0.997, 95% CI 0.995 to 1.000, *p* = 0.058) with Aβ positivity. In the analyses with all cholesterol type (LDL-c, HDL-c, and triglyceride) together as predictors, the results showed a similar trend. Specifically, LDL-c levels (β = 0.071, *p* = 0.001) were independently associated with Aβ uptake, while HDL-c (β = 0.004, *p* = 0.879) and triglycerides levels (β =  − 0.036, *p* = 0.179) were not associated with Aβ uptake. HDL-c levels (β =  − 0.071, *p* = 0.039) were negatively associated with WMH volume, while LDL-c (β = 0.040, *p* = 0.221) and triglyceride (β =  − 0.009, *p* = 0.781) levels were not associated with WMH volume. LDL-c levels (β =  − 0.067, *p* = 0.013) were negatively associated with hippocampal volume, whereas HDL-c (β = 0.018, *p* = 0.538) and triglyceride (β = 0.013, *p* = 0.640) levels were not associated with hippocampal volume.

## Discussion

In the present study, we systematically investigated the relationships between plasma cholesterol profile components and biomarkers of AD and CSVD in a large non-demented Korean cohort. Our major findings are as follows: First, increased LDL-c levels were associated with greater Aβ uptake, independent of *APOE4* genotype and lipid-lowering medication. Second, decreased HDL-c levels were predictive of higher WMH volumes. Finally, increased LDL-c levels were associated with a decreased hippocampal volume, which was partially mediated by Aβ uptake. Taken together, our findings suggest that increased LDL-c and decreased HDL-c levels are important risk factors for AD and CSVD, respectively. Furthermore, considering that plasma cholesterol profile components are potentially modified by diet, exercise, and pharmacological agents, our results provide evidence that regulating LDL-c and HDL-c levels may be a strategy to prevent dementia.

Our first major finding was that increased LDL-c levels were associated with increased Aβ uptake, independent of *APOE4* genotype and lipid-lowering medication. Studies based on NHWs have shown inconsistent results regarding the association between hypercholesterolemia and Aβ uptake. Specifically, previous studies showed that increased LDL-c levels were associated with increased Aβ uptake [7, 25]. In contrast, another study did not show any relationship between the plasma cholesterol profile components and Aβ uptake [9]. However, the present study was based on the Korean population, and the sample size was the largest among studies investigating the effects of plasma cholesterol on Aβ uptake. Thus, our findings demonstrated that increased LDL-c levels were an important risk factor for increased Aβ uptake in non-demented participants, at least in the Korean population. However, unlike a previous study [7], we did not find any relationship between decreased HDL-c levels and Aβ uptake. One study suggested that decreased HDL-c levels are associated with increased Aβ uptake.

However, the potential pathophysiological role of LDL-c in Aβ uptake remains unclear. Although cholesterol in the brain plays important roles in Aβ synthesis and clearance, its bioactivity in the brain is independent of plasma cholesterol because of the blood–brain barrier (BBB). However, some studies have shown that plasma cholesterol levels are closely associated with BBB permeability [26–28]. Specifically, systemic hypercholesterolemia may damage the BBB via inflammation and other mechanisms, with consequent leakage of serum cholesterol, inflammatory cytokines, and amyloidogenic factors. Circulating cholesterol levels through BBB damage may lead to the destruction of cholesterol homeostasis in the brain, which may lead to AD pathology. In particular, LDL-c activates microvascular endothelial cells to increase the secretion of inflammatory mediators such as TNF-α and IL-6. LDL-c also mediates the decreased expression of tight junction protein ZO-1, indicating that LDL-c contributes to the increased permeability of the BBB in AD [29]. Conversely, there might be possibility that cholesterol metabolism is affected by Aβ uptake. Recent studies have found that Aβ have a deleterious effect on proteins involved in cholesterol metabolism including 3-hydroxy-3-methyl-glutaryl-coenzyme A reductase and ATP-binding cassette transporter A1 [30].

Our second major finding was that decreased HDL-c levels were independently associated with increased WMH volume. This inverse association is consistent with the results of previous studies [31–34], which indicated that lower HDL-c levels were associated with higher WMH severity. HDL is well-known for its vasoprotective function via several mechanisms, such as reverse cholesterol transport, which removes cholesterol from lipid-laden macrophages in the vessel wall [35]. Additionally, HDL reduces neuroinflammation and increases bioavailability of endothelial nitric oxide synthase [36, 37]. Neuroinflammation and neurovascular decoupling caused by the impairment of endothelial nitric oxide synthase lead to CSVD burden [38].

In the present study, we found no relationship between triglyceride and imaging markers of AD and CSVD. Although triglyceride is another major cholesterol marker for hypercholesterolemia, results regarding the relationship between triglyceride and the development of dementia are inconclusive. A long-term follow-up longitudinal study revealed that increased triglyceride levels increase the risk of dementia [39]. However, another study showed no significant difference in triglyceride levels between patients with AD and cognitively unimpaired elderly individuals [40]. In fact, similar to our results, a pathological study found no association between triglyceride levels and AD pathological burden [25].

Our final major finding was that increased LDL-c levels were associated with decreased hippocampal volume, which was partially mediated by Aβ uptake. Previous results regarding the association between LDL-c and neurodegeneration have been somewhat inconsistent [41, 42]. However, considering that increased Aβ uptake was predictive of hippocampal atrophy [43], the mediating effects of Aβ uptake on the relationship between LDL-c levels and hippocampal atrophy observed in the present study could be explained. We also found that increased LDL-c levels were associated with decreased hippocampal atrophy, without the mediation of Aβ uptake. Given that LDL-c is a risk factor for arteriosclerosis and that the hippocampus is also vulnerable to cerebral ischemia, recurrent vascular insult associated with LDL-c may lead to hippocampal atrophy. Aβ-independent tauopathy may also be a possible mechanism underlying these findings. In fact, a recent study found that hypercholesterolemia directly affects tau deposition irrespective of Aβ uptake, which leads to hippocampal atrophy [42].

### Limitations

The strength of the present study was that we systematically investigated the association of each cholesterol profile component with Aβ uptake and WMH volume in a large Korean cohort, after controlling for various potential confounding factors. However, our study had several limitations that need to be addressed. First, our major limitation is that our findings were cross-sectional and obtained later in life. Thus, the temporal relationship between LDL-c and Aβ uptake could not be determined because of the intrinsic limitations of a cross-sectional study. Second, the generalizability of the present study to community-based populations needs to be treated with caution because the cohort was recruited from a memory clinic setting, which results in the enrollment of a more “health-seeking” population. Third, the effect of LDL-c on Aβ uptake was relatively modest. However, given the paucity of modifiable risk factors for the development of Aβ, pharmacological agents targeting LDL-c might be clinically useful to prevent Aβ deposition. Nevertheless, our study is relevant as it provides a comprehensive understanding of the relationship between each cholesterol profile component and imaging biomarkers of AD and CSVD and their downstream markers.

## Conclusions

In conclusion, increased LDL-c and decreased HDL-c levels were important risk factors for AD and CSVD, respectively. Furthermore, increased LDL-c levels are predictive of hippocampal atrophy related to Aβ uptake. Therefore, lowering LDL-c and increasing HDL-c levels may reduce Aβ uptake and CSVD burden, respectively, eventually resulting in a delay in cognitive decline.

## Data Availability

Anonymized data for our analyses presented in the present report are available upon request from the corresponding authors.
